# Age dependence of tumor genetics in unfavorable neuroblastoma: arrayCGH profiles of 34 consecutive cases, using a Swedish 25-year neuroblastoma cohort for validation

**DOI:** 10.1186/1471-2407-13-231

**Published:** 2013-05-09

**Authors:** Cihan Cetinkaya, Tommy Martinsson, Johanna Sandgren, Catarina Träger, Per Kogner, Jan Dumanski, Teresita Díaz de Ståhl, Fredrik Hedborg

**Affiliations:** 1 Department of Immunology, Genetics and Pathology, Rudbeck Laboratory, Uppsala University, Uppsala SE-751 85, Sweden; 2 Department of Surgical Sciences, Endocrine Unit, Uppsala University, University Hospital, Uppsala, SE-751 85, Sweden; 3 Department of Clinical Genetics, Institute of Biomedicine, University of Gothenburg, Sahlgrenska Hospital, Göteborg, SE-413 45, Sweden; 4 Department of Oncology-Pathology, Cancer Center Karolinska, CCK R8:04, Karolinska Institutet, Stockholm, SE-171 76,, Sweden; 5 Department of Women’s and Children’s Health, Childhood Cancer Research Unit, Karolinska Institutet, Karolinska Hospital, Stockholm, SE 171 76, Sweden; 6 Department of Women’s and Children’s Health, Uppsala University, University Hospital, Uppsala SE-751 85, Sweden

**Keywords:** High-risk, Unfavorable, Neuroblastoma, Arraycgh, DNA copy number, Gain, Loss, Amplification, Age

## Abstract

**Background:**

Aggressive neuroblastoma remains a significant cause of childhood cancer death despite current intensive multimodal treatment protocols. The purpose of the present work was to characterize the genetic and clinical diversity of such tumors by high resolution arrayCGH profiling.

**Methods:**

Based on a 32K BAC whole-genome tiling path array and using 50-250K Affymetrix SNP array platforms for verification, DNA copy number profiles were generated for 34 consecutive high-risk or lethal outcome neuroblastomas. In addition, age and *MYCN* amplification (MNA) status were retrieved for 112 unfavorable neuroblastomas of the Swedish Childhood Cancer Registry, representing a 25-year neuroblastoma cohort of Sweden, here used for validation of the findings. Statistical tests used were: Fisher’s exact test, Bayes moderated t-test, independent samples t-test, and correlation analysis.

**Results:**

MNA or segmental 11q loss (11q-) was found in 28/34 tumors. With two exceptions, these aberrations were mutually exclusive. Children with MNA tumors were diagnosed at significantly younger ages than those with 11q- tumors (mean: 27.4 *vs.* 69.5 months; p=0.008; n=14/12), and MNA tumors had significantly fewer segmental chromosomal aberrations (mean: 5.5 *vs.* 12.0; p<0.001). Furthermore, in the 11q- tumor group a positive correlation was seen between the number of segmental aberrations and the age at diagnosis (Pearson Correlation 0.606; p=0.037). Among nonMNA/non11q- tumors (n=6), one tumor displayed amplicons on 11q and 12q and three others bore evidence of progression from low-risk tumors due to retrospective evidence of disease six years before diagnosis, or due to tumor profiles with high proportions of numerical chromosomal aberrations. An early age at diagnosis of MNA neuroblastomas was verified by registry data, with an average of 29.2 months for 43 cases that were not included in the present study.

**Conclusion:**

MNA and segmental 11q loss define two major genetic variants of unfavorable neuroblastoma with apparent differences in their pace of tumor evolution and in genomic integrity. Other possible, but less common, routes in the development of aggressive tumors are progression of low-risk infant-type lesions, and gene amplifications other than *MYCN*. Knowledge on such nosological diversity of aggressive neuroblastoma might influence future strategies for therapy.

## Background

Neuroblastoma is a childhood malignancy that arises from embryonic cells of the sympathetic ganglia or the adrenal medulla [1]. It is mainly a disease of infants and toddlers; more than half of patients with neuroblastoma are diagnosed before two years of age and ~90 percent before age six [2,3]. This age dependence of the incidence of neuroblastoma may be a consequence of developmentally determined disappearance of the pool of immature cells from which neuroblastomas are thought to derive. The disease is clinically diverse, and ranges from cases with a very dismal prognosis, despite modern intensive multimodal therapy, to those with an excellent chance of survival [2]. This variation in clinical behavior is also highly age dependent: children who are diagnosed after two years of age suffer predominantly from aggressive forms. Neuroblastomas that are diagnosed during adolescence and young adulthood are rare, but they are of particular concern because they almost invariably progress, although with an indolent course [4,5]. In sharp contrast, tumors that are diagnosed before 18 months of age are generally associated with a favorable prognosis. Such tumors are usually less advanced, have a propensity for spontaneous involution or maturation, and respond well to mild chemotherapy [2].

These clinical differences correspond to clear differences in tumor genetics [2]. As a general rule, prognostically favorable tumors display numerical imbalances of entire chromosomes and have near-triploid DNA content, whereas higher-risk tumors present with segmental chromosomal aberrations (SCAs) and are often pseudo-diploid. *MYCN* gene amplification (MNA) was one of the first genetic markers for highly aggressive neuroblastoma to be established [6], and remains a powerful prognostic indicator [7]. More recently, an independent prognostic value of a segmental deletion of 11q has also been recognized [7-9]. Both these aberrations are incorporated in the present International Neuroblastoma Risk Group (INRG) classification system for treatment stratification [10]. Several arrayCGH studies in the recent years support the MNA/segmental 11q loss dichotomy of high-risk neuroblastoma and indicate that any type of segmental numerical chromosomal aberration is a negative prognostic sign [11-13]. However, the representativeness of the studied tumor materials may be questioned because the tumors were from multiple sources and, hence, selection bias may have occurred. Therefore, the present study aims at characterizing the heterogeneity of genetic aberrations in aggressive neuroblastoma by exploiting a consecutive, population-based series of tumors, the representativeness of which was tested against data in the Swedish Childhood Cancer Registry. The most striking observations were related to age at tumor presentation: MNA tumors were associated with a particularly early age at diagnosis and low numbers of other chromosomal aberrations suggesting a rapid tumor evolution with few genetic hits involved, whereas 11q deleted tumors were diagnosed at older ages and showed significantly more SCAs, the numbers of which were positively correlated with the age at diagnosis, suggesting a chromosomal instability phenotype with a more stepwise tumor evolution. Other tumors seemed to be the result of late progression of low-risk neuroblastoma or of gene amplifications other than *MYCN*. This clinicogenetic diversity of unfavorable neuroblastoma is likely to reflect differences in tumor evolution and growth, which may have therapeutic implications.

## Methods

### Study design

In order to obtain a representative view at high resolution of DNA copy number aberrations in aggressive forms of neuroblastoma a 32K BAC whole-genome tiling path arrayCGH platform was applied to a consecutive, population-based tumor material (described below). The representativeness of the tumor collection was analyzed by comparing the patients’ ages at diagnosis and proportions of tumors in relation to the presence or absence of MNA with the corresponding data of neuroblastomas registered in the Swedish Childhood Cancer Registry during a 25-year period. For verification of the BACarray-based profiles high-resolution SNP array analyses were performed. Based on publically available gene expression data from neuroblastoma, expression profiles were compared between tumor groups for certain chromosomal regions of interest.

### Patient material

Fresh frozen specimens of neuroblastoma were collected consecutively during the period 1986–1994 at all Swedish centers at which pediatric tumor surgery is performed [14]. Samples collected between 1995 and 2010 at Uppsala University Hospital, which treats approximately 20 percent of Swedish patients with neuroblastoma, were also included. The inclusion criteria were: high-risk neuroblastoma, as defined by the INRG classification system [10], progression to disseminated fatal disease, and stage L2 tumors in children >12 years of age at diagnosis (one case). The INRG high-risk criteria applied here were: Stage M tumors in children >18 months of age at diagnosis and all tumors with MNA. Stage MS tumors were excluded. The individual clinical data of all 34 cases included in the study are shown in Table 1.

**Table 1:** Clinical data and main genetic findings of 34 unfavorable neuroblastomas

**ID**	**Age**	**Sex**	**Stage**	**Outcome**	**Followup**	**Survival median**	**Site**	**WCA**	**WCA**	**SCA**	**SCA**	**MNA**	**11q-**	**Array platform**
	**(mo)**		**(INRGSS)**		**(mo)**	**(mo)**		**(nr)**	**(average)**	**(nr)**	**(average)**			
52*	4	m	M	DOD	10		adr	0		6		+		32K
55	8	f	M	DOD	16		adr	0		6		+		32K, Affymetrix
106*	10	f	M	NED	265		adr	0		12		+		32K
123*	11	f	M	DOD	3		adr	0		6		+		32K, Affymetrix
241*	11	m	M	DOD	8		adr	1		3		+		32K, Affymetrix
244	14	f	M	DOD	22		adr	8		2		+		Affymetrix
212	15	m	M	DOD	5		adr	0		2		+		32K, Affymetrix
240*	21	f	M	NED	43		adr	0		3		+		32K, Affymetrix
135*	22	m	L2	DOD	3		adr	0		3		+		32K, Affymetrix
95*	26	f	M	DOD	15		adr	0		8		+		32K
238	30	f	M	DOD	24		adr	0		6		+		Affymetrix
207	37	f	M	DOD	7		adr	2		5		+		32K, Affymetrix
217	37	m	M	DOD	36		adr	0		8		+		32K
126	138	m	M	DOD	9		adr	0		7		+		32K, Affymetrix
**MNA**^**not11q-**^						**9.5**			**0.8**		**5.5**			
68	41	m	M	DOD	12		adr	9		7		+	+	32K, Affymetrix
136*	48	f	L2	DOD	12		adr	1		8		+	+	32K
**MNA** &**11q-**						**12.0**			**5.0**		**7.5**			
243	32	f	M	DOD	28		adr	2		10			+	Affymetrix
149	34	m	M	DOD	16		adr	0		12			+	32K, Affymetrix
112	40	m	M	DOD	18		adr	1		3			+	32K, Affymetrix
111*	42	f	M	NED	262		adr	0		7			+	32K
155	52	f	M	DOD	19		adr	3		13			+	32K, Affymetrix
32*	57	m	M	DOD	12		adr	2		15			+	32K, Affymetrix
110	60	m	M	DOD	12		adr	1		14			+	32K, Affymetrix
69*	60	m	L2	DOD	17		th	1		14			+	32K, Affymetrix
41*	77	f	M	DOD	8		th	2		15			+	32K, Affymetrix
49	82	m	M	DOD	14		adr	3		10			+	32K, Affymetrix
209	129	m	M	DOD	35		adr	2		10			+	32K, Affymetrix
229	169	m	L2	DOD	34		adr	1		21			+	32K, Affymetrix
**11q-**^**notMNA**^						**16.5**			**1.5**		**12.0**			
107*	23	m	M	DOD	10		adr	1		2				32K, Affymetriix
131	37	f	L2	DOD	21		adr	13		1				32K, Affymetriix
130	46	m	M	DOD	57		adr	0		3				32K, Affymetriix
208	59	m	L2	DOD	25		renal	4		3				32K
242*	90	f	M	SD	37		th	12		5				32K, Affymetriix
226*	91	m	M	SD	61		th	2		12				32K, Affymetriix
**Not MNA not 11q-**						**23.0**			**5.3**		**4.3**			

Cases are sorted on the basis of genetic category, as determined by the presence of MNA and segmental loss of 11q. Within each tumor category, cases are sorted according to age at diagnosis. Abbreviations: DOD: dead of disease; NED: no evidence of disease; SD: stable disease; WCA: whole-chromosome copy number aberration; SCA: segmental chromosomal copy number aberration; adr: adrenal; th: thoracic. Tumors marked with asterisk (*) with IDs: 52, 106, 123, 241, 240, 135, 95, 136, 111, 32, 69, 41, 107, 242, and 226 are reported also by Carén et al. [15] with the respective codes: 7, 14, 8, 2, 4, 13, 12, 37, 40, 42, 44, 39, 66, 73, and 63, as listed in [15; Table S1].

To ensure that the tumor specimens represented viable tumor tissue their quality was assessed from hematoxylin/eosin stained cryosections, requiring a tumor cell content of at least 60–70%. Ethical approval was obtained from the Regional Ethical Review Board in Uppsala (approval 2007/069), and written informed consent was obtained from the parents.

There is an overlap between tumors included in this work and those of a similar Swedish report [15]. However, our study is based on another collection of biopsies from a partially different set of tumors. The previously reported Affymetrix SNParray data [15] was used for verification of our BACarray results on tumors common to both studies (n=15) and for verification of our data on presently unique tumors (n=19) new original SNParray data was produced. Tumors in common with the aforementioned study are indicated in Table 1 and information on their previous codes [15; Table S1] is given in the table legend.

### Array-based comparative genomic hybridization

The 32K BAC array was established as reported previously [16]. High-quality DNA was obtained by standard methods [17]. DNA labeling, hybridization, washing, scanning of arrays, and data processing were performed as described earlier [16,18-20]. Experiments using 50K and 250K Affymetrix arrays were performed in accordance with the manufacturer’s protocol (Affymetrix, Inc., Santa Clara, CA), and as described earlier [21].

### Microarray expression data

Publically available gene expression data from high-risk metastatic neuroblastomas, series GSE13136 [22], platform Affymetrix Human Genome U133 Plus 2.0, which were selected by the presence of MNA (GSM328993, GSM328996, GSM329000, GSM329006, GSM329007, GSM329008, GSM329011, GSM329012, GSM329013, GSM329015) or segmental 11q loss (GSM328992, GSM328995, GSM328997, GSM328999, GSM329002, GSM329010, GSM329014, GSM329017) were downloaded from Gene Expression Omnibus (http://www.ncbi.nlm.nih.gov/geo/) and normalized in Expression Console v1.1 (3' Expression Arrays-RMA, Affymetrix).

### The Swedish childhood cancer database

Clinical data and the *MYCN* copy number status for neuroblastomas diagnosed in Sweden during the 25-year period of 1984–2008 were obtained from the Swedish Childhood Cancer Registry. The clinical criteria for inclusion were the same as for the array study. The limit for MNA was set at >4 copies of *MYCN* per haploid genome, as determined by FISH and/or SNParray.

### Statistical analysis

To analyze differences in DNA copy number among the tumor groups, Fisher’s exact test was used within the Nexus Copy Number 5.0 analysis program (BioDiscovery, Inc., El Segundo, CA, USA). To search for genes that were differentially expressed among the tumor groups, an empirical Bayes moderated *t*-test was applied using the ‘limma’ package [23] and p-values were adjusted in accordance with the method of Benjamini and Hochberg [24]. Clinical data were processed using PASW Statistics 18.0 software (SPSS; Chicago, IL, USA). Mean differences in age were examined with the *t*-test for independent samples. Co-variations were analyzed by correlation analysis, and the results were expressed as Pearson correlation coefficients.

## Results

### Identification of two major unfavorable neuroblastoma groups with different genomic signatures

**Figure 1:**
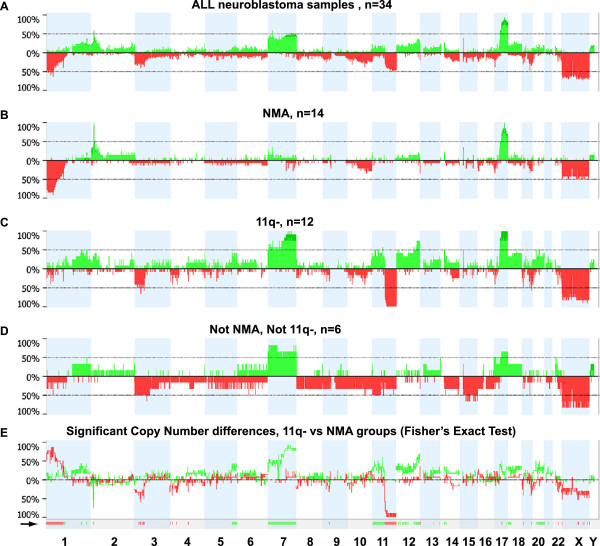
**Genetic findings in unfavorable neuroblastoma. **The frequency of copy number changes was calculated for all measurement points in the arrays and plotted relative to the position along the chromosome for: (**A**): all tumors, (**B**): MNA^not11q- ^tumors, (**C**): 11q-^notMNA ^tumors, (**D**): neither MNA nor 11q loss tumors. The number of analyzed tumors is indicated (n). Green bars above the horizontal line indicate the percentage of tumors with copy gains and red bars below the horizontal line indicate the percentage of tumors with copy losses. Data for the X chromosome were normalized to female reference DNA and the respective proportion of boys in panels A-D were: 56%, 43%, 67%, and 67%, respectively (**E**): To search for copy number alterations that differ between the11q-^notMNA ^and MNA^not11q- ^groups, the frequency percentage *difference *between the two groups are plotted: *Copy number gain difference *(green graph): Values above baseline represent regions in which gains are more numerous among 11q-^not MNA^ tumors, and vice versa for values below baseline. *Deletion difference *(red graph): Values above baseline represent regions in which losses are more common among MNA^not11q- ^tumors, and vice versa for values below baseline. The regions significantly differentially altered between the groups, identified by using Fisher's exact test within Nexus copy-number software, (p<0.05 and threshold difference in frequency >25%), are shown below the graph, as indicated by a black arrow.

**Figure 2:**
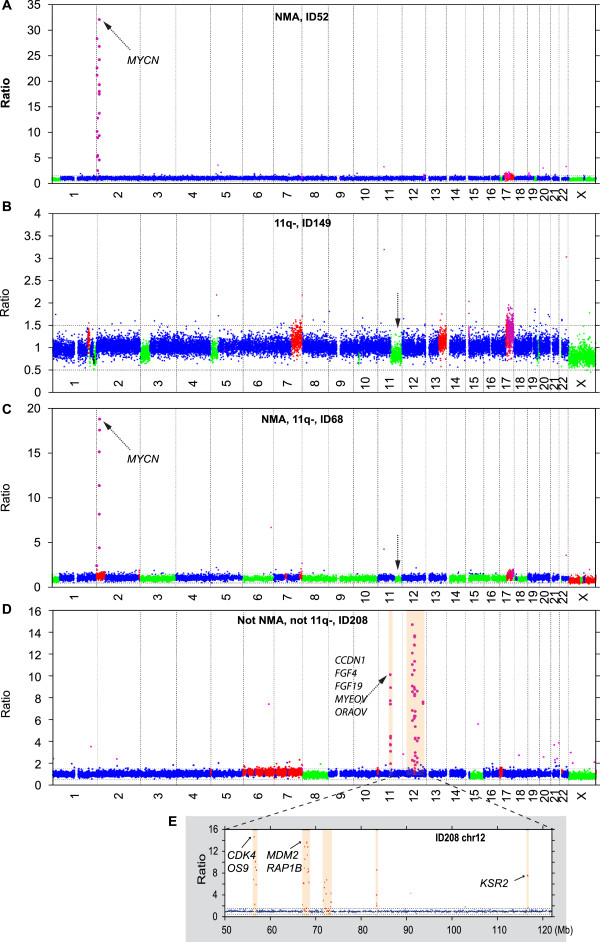
**Examples of individual neuroblastoma profiles within genetic subgroups. **(**A**) MNA^not11q-^; (**B**) 11q-^notMNA^; (**C**) combined MNA and segmental 11q loss; (**D**) neither MNA nor segmental 11q loss; (**E**) shows an expanded segment of chromosome 12 in panel (D). Amplified genes of particular oncogenic interest are indicated. Each individual clone was assigned a copy number class as follows: *i) *balanced: two alleles (blue dots); *ii) *gained: presence of three (red) or more (pink dots) alleles; or *iii) *deleted: hemizygous deletions (green dots). No homozygous deletions were found in these tumors. Black arrows indicate MNA amplification, 11q loss or other amplifications.

To visualize the results from the complete set of tumors, the percentages of tumors with copy number change were calculated and plotted relative to the position along the chromosomes (Figure 1A). All individual profiles are also illustrated (Additional file 1: Figure S1). Partial or complete gain of one or two copies of the 17q arm was the most common aberration (88% of the tumors), followed by loss of 1p segments (56%), MNA (47%), and loss of 11q (47%; 14 tumors with segmental loss and 2 with loss of one entire chromosome 11) (Figure 1A and Additional file 1: Figure S1). Subsequently, we examined the frequencies of copy number changes in tumor subgroups that were defined on the basis of the absence or presence of MNA and segmental 11q loss (11q-); hence, the tumors were separated into four subgroups: MNA^not11q-^ (n=14), 11q-^notMNA^ (n=12), MNA and 11q- (n=2), and neither MNA nor 11q- (n=6). The results are shown in Figure 1B-D and Additional file 1: Figure S1. Selected profiles from each group are shown in Figure 2. Analysis using Fisher’s exact test of differences between the MNA^not11q-^ and 11q-^notMNA^ groups (which contained most of the samples, 26/34; 76%) revealed that loci on 1p, 2p, 3p, 5q, 7, 11, 12, 18p, and 20q were differentially altered between the two sets of tumors (Figure 1E). These groups also differed in terms of the number of SCAs (mean 5.5 and 12.0, respectively; Table 1, p<0.001, Wilcoxon’s test), and with regard to an absence of numerical whole chromosomal aberrations, which was the case in 11/14 MNA^not11q-^ tumors, but in only 2/12 11q-^notMNA^ tumors (Table 1 and Additional file 1: Figure S1). Finally, tumors that showed neither MNA nor 11q- were more heterogeneous in terms of both segmental and whole chromosomal aberrations; two tumors showed aberrations in chromosome number for the majority of chromosomes (ID 131 and ID 242; Table 1 and Additional file 1: Figure S1).

### Age dependence of genetic subgroups

**Figure 3:**
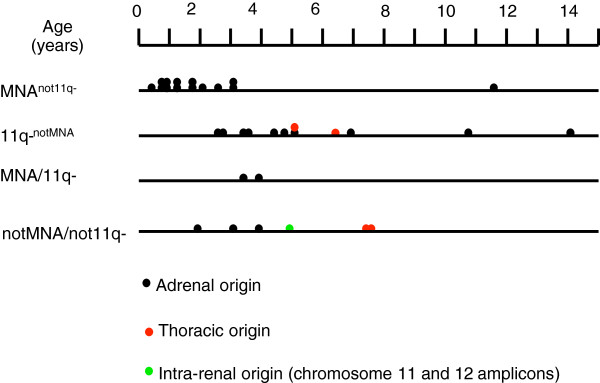
**Age at diagnosis in unfavorable neuroblastoma (n=34). **Each tumor in the present study is plotted on a time axis according to age at diagnosis and genetic subgroup. Color symbols indicate the likely site of origin.

As shown in Table 1 and in Figure 3, MNA tumors were diagnosed early in life. In fact, 11 out of the 12 children who were youngest at diagnosis suffered from MNA tumors. If an outlier amongst the MNA group in terms of genetic profile and age (ID126; Table 1 and Additional file 1: Figure S1) is disregarded, only two 11q-^notMNA^ tumors were diagnosed in the same young age range as that of the remaining MNA^not11q-^ tumors (Table 1 and Figure 3). Statistically, the ages at diagnosis of children with MNA^not11q-^ tumors differed highly significantly from those of children with 11q-^notMNA^ tumors (mean age: 27.4 *vs.* 69.5 months, respectively; p=0.008; median age: 18 *vs.* 58.5 months, respectively; n=14 *vs.* 12).

To test the validity of these findings in a larger sample, we utilized the Swedish Childhood Cancer Registry, which contained clinical information and data on the copy number of *MYCN* in tumors for 240 cases of neuroblastoma that were diagnosed during almost the same time period as the cases in the present study. Among these, 112 were unfavorable cases (100 high risk and 12 intermediate risk who suffered lethal tumor progression). Of the tumors included in the current study, 25 were represented among these 112 cases in the registry, and 11 of the 25 were cases with MNA. Given that 11q status is not registered consistently in the database, we compared the age at diagnosis of children with MNA tumors to those with non-MNA tumors. This analysis revealed highly significant differences both for the present study (MNA *vs.* non-MNA: mean: 29.5 *vs.* 65.6 months; p=0.005; median age: 21 *vs.* 58 months; n=16 *vs.* 18) and for cases of unfavorable neuroblastoma in the registry that were not included in the present study (MNA *vs.* non-MNA: mean age: 29.2 *vs.* 49.1 months; p=0.001; median age: 24 *vs.* 43.5 months; n=43 *vs.* 44). To determine whether the MNA cases included in our study might be biased towards a younger age range, we compared their ages at diagnosis to those of the other MNA cases in the registry and found no statistical difference (mean: 29.5 *vs.* 29.2 months; median: 21 *vs.* 24 months; p=0.968; n=16 *vs.* 43). There was also no statistically significant difference in the distribution of ages at diagnosis for non-MNA tumors (mean: 65.6 *vs.* 49.1 months; median: 58 *vs.* 43.5 months; p=0.098; n=18 *vs.* 44).

**Figure 4:**
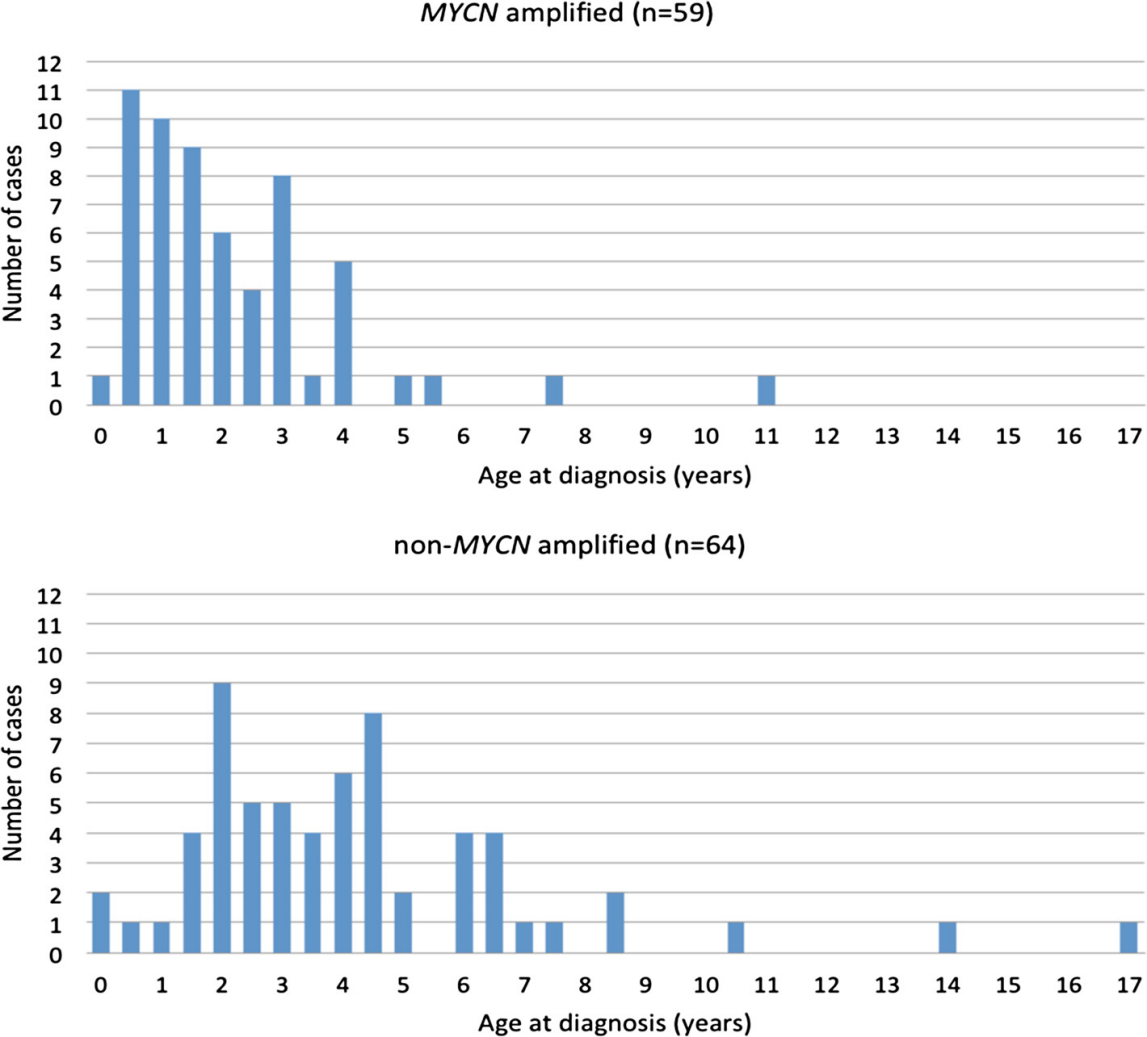
***MYCN *amplification status and age at diagnosis of Swedish patients with unfavorable neuroblastoma (n=121). **Present cases, representing the period 1986–2010, have been merged with all other cases of unfavorable neuroblastoma found in the Swedish Childhood Cancer Registry during the period 1984–2008. Data is presented in 6-month age intervals. Unfavorable criteria were: lethal tumor progression, *MYCN* amplification, INRGSS Stage M and >18 months of age at diagnosis, and INRGSS Stage L2 >12 years of age at diagnosis.

When the findings were merged, we were able to conclude that, among Swedish children who were diagnosed with unfavorable neuroblastoma during this time period, MNA tumors were almost four-fold more common than non-MNA tumors when diagnosis was made before two years of age (31 *vs.* 8), whereas this relationship was reversed in children who were diagnosed after 3.5 years of age (10 *vs.* 35; Figure 4).

**Figure 5:**
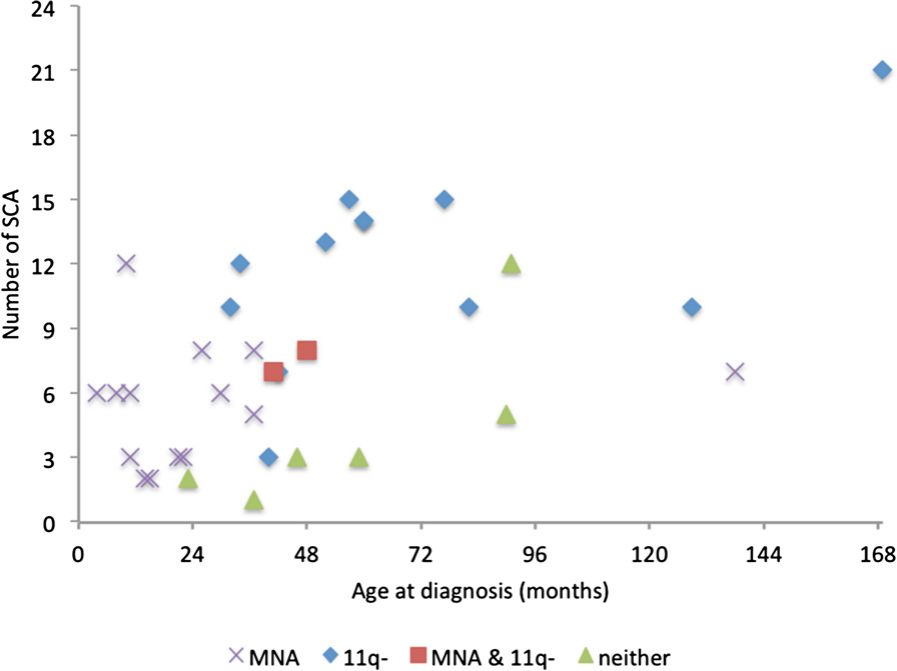
**Age dependence of segmental chromosomal aberrations in unfavorable neuroblastoma (n=34). **Data are separated by genetic subtype, as indicated. X-axis: age at diagnosis (years). Y-axis: number of segmental chromosomal aberrations (SCA; amplicons not included).

In view of the high number of SCAs that were found in tumors with segmental 11q deletion, we investigated the possibility of an age-dependence for the number of SCAs within this tumor subgroup and found a positive correlation with age at diagnosis (Pearson correlation 0.606; p=0.037). When merging the four genetic groups the age dependence of SCA numbers was even more evident with a p-value of 0.001 (Pearson correlation 0.547; Figure 5).

### High copy number amplicons

In total, 17 tumors displayed amplified regions. The number of these amplified regions per tumor varied from one to seven, and their sizes ranged from 0.15 to 6.8 Mb. Of the 16 tumors with MNA, either the *MYCN*-containing amplicon was the only amplification event or it was accompanied by multiple amplified loci within 2p, and in one case by two amplified loci on chromosome 3q. The regions of amplification, their frequencies, and the genes encompassed are listed in Table 2. In one tumor with MNA and its associated cell line [15], a few novel amplicons were found, which encompassed genes such as *GDF7*, *FSHR*, *PRKCE*, and *TMEM18* (Additional file 2: Figure S2). Overall, 20% of the amplified loci did not encompass any gene (Table 2). One unusual case (ID 208) displayed multiple amplicons but not MNA. Genes of particular oncogenic interest within these loci were *CCND1*, *FGF4*, *FGF19*, *IGHMBP2, MYEOV*, and *ORAOV1* on 11q13.2-q13.3 and *CDK4*, *MDM2*, and *KSR2* on 12q13.3-q15 (Figure 2D-E and Table 2).

**Table 2:** **Regions of amplification in unfavorable neuroblastoma** (n=17)

**Chr Region (Mb)**	**Cyto-band**	**Length(Mb)**	**Genes**	**Gene Symbols**	**Group**	**No of tumors**
chr:2:0.366-0.793	p25.3	0.427	1	*TMEM18*	NMA	1
chr:2:2.304-6.219	p25.3-p25.2	3.915	12	*ADI1, ALLC, COLEC11, LOC150622, LOC400940, LOC730811, MYT1L, RNASEH1, RPS7, SOX11, TSSC1, TTC15*	NMA	1
chr:2:3.605-4.280	p25.3	0.675	3	*ALLC, COLEC11, RPS7*	NMA	1
chr:2:5.708-7.439	p25.2-p25.1	1.731	6	*CMPK2, LOC150622, LOC400940, RNF144A, RSAD2, SOX11*	NMA	1
chr:2:6.372-6.855	p25.2	0.483	0		NMA	1
chr:2:10.413-11.244	p25.1	0.831	10	*ATP6V1C2, C2orf50, HPCAL1, KCNF1, NOL10, ****ODC1****, PDIA6, PQLC3, ROCK2, SNORA80B*	NMA	2
chr:2:11.622-11.878	p25.1	0.256	3	*GREB1, LPIN1, NTSR2*	NMA	1
chr:2:13.097-13.580	p24.3	0.483	0		NMA	1
chr:2:14.219-14.292	p24.3	0.073	0		NMA	2
chr:2:15.895-16.095	p24.3	0.200	2	***MYCN****, MYCNOS*	NMA	16
chr:2:16.675-16.705	p24.3	0.030	1	*FAM49A*	NMA	6
chr:2:16.846-17.106	p24.3-p24.2	0.260	0		NMA	6
chr:2:18.197-18.423	p24.2	0.227	0		NMA	3
chr:2:20.546-21.012	p24.1	0.466	3	*C2orf43, GDF7, HS1BP3*	NMA	1
chr:2:22.493-25.674	p24.1-p23.3	3.181	21	*ADCY3, ATAD2B, C2orf44, C2orf79, C2orf84, CENPO, DNAJC27, DNMT3A, DTNB, EFR3B, FKBP1B, ITSN2, KLHL29, LOC375190, MFSD2B, NCOA1, PFN4, POMC, SF3B14, TP53I3, UBXN2A*	NMA	1
chr:2:26.853-27.169	p23.3	0.316	9	*AGBL5, C2orf18, CENPA, DPYSL5, EMILIN1, KHK, LOC100128731, MAPRE3, TMEM214*	NMA	2
chr:2:28.022-28.430	p23.2	0.408	1	*BRE*	NMA	2
chr:2:29.071-30.833	p23.2-p23.1	1.762	8	***ALK****, C2orf71, CAPN13, CLIP4, FAM179A, LBH, LCLAT1, YPEL5*	NMA	2
chr:2:38.841-39.010	p22.1	0.168	4	*DHX57, GEMIN6, LOC100271715, MORN2*	NMA	1
chr:2:45.742-46.467	p21	0.725	2	*EPAS1, PRKCE*	NMA	1
chr:2:47.379-47.698	p21-p16.3	0.319	3	*EPCAM, KCNK12, MSH2*	NMA	1
chr:2:48.848-49.512	p16.3	0.664	1	*FSHR*	NMA	1
chr:3:170.768-172.093	q26.2	1.325	18	*ARPM1, CLDN11, EIF5A2, GPR160, LOC100128164, LRRC31, LRRC34, LRRIQ4, MECOM, MYNN, PHC3, ****PRKCI****, RPL22L1, SAMD7, SEC62, ****SKIL****, SLC7A14, TERC*	NMA	1
chr:3:173.047-173.459	q26.31	0.411	2	*FNDC3B, TMEM212*	NMA	1
chr:11:68.463-69.308	q13.2-q13.3	0.845	9	***CCND1****, FGF19, ****FGF4****, IGHMBP2, MRGPRD, MRGPRF, MYEOV, ORAOV1, TPCN2*	Not NMA, not 11q-	1
chr:12:56.182-57.066	q13.3-q14.1	0.884	23	*AGAP2, AVIL, B4GALNT1, ****CDK4****, CTDSP2, CYP27B1, DCTN2, DDIT3, DTX3, FAM119B, GEFT, KIF5A, LOC100130776, MARCH9, MARS, MBD6, METTL1, OS9, PIP4K2C, SLC26A10, TSFM, TSPAN31, XRCC6BP1*	Not NMA, not 11q-	1
chr:12:67.060-68.692	q15	1.632	13	*BEST3, CCT2, CPM, CPSF6, FRS2, LRRC10, LYZ, ****MDM2****, NUP107, RAB3IP, ****RAP1B****, SLC35E3, YEATS4*	Not NMA, not 11q-	1
chr:12:71.578-73.413	q21.1	1.835	1	*LOC552889*	Not NMA, not 11q-	1
chr:12:83.287-83.563	q21.31	0.276	0		Not NMA, not 11q-	1
chr:12:116.476-116.802	q24.22-q24.23	0.326	1	*KSR2*	Not NMA, not 11q-	1

Regions that involved at least two neighboring clones, with copy number count >3 and normalized fluorescence ratio >2 are shown. For amplicons with regions shared between tumors, the minimal overlapping region is shown. Genes of particular oncogenic interest in neuroblastoma are indicated in bold.

### Differentially expressed genes within aberrant regions of MNA and 11q-deleted tumors

Given that MNA and 11q- neuroblastomas present with divergent genomic signatures, we sought differences in gene expression profiles within the regions that differed most consistently between these two groups (1p, 2p, and chromosomes 7 and 11). For this purpose, we compared publicly available gene expression data for high-risk neuroblastomas with recorded MNA and 11q status (see Methods). Among the MNA tumors (n=10), five tumor suppressor genes (among other genes) were underexpressed within the distal 1p: *CAMTA1*, *KIF1B*, *PRDM2*, *FABP3*, and *CDKN2C*; whereas *MYCNOS* and *MYCN* were the two top differentially upregulated genes on 2p. Several constituents of the extracellular matrix or membrane proteins involved in cell adhesion, motility or proliferation that map to chromosome 7, namely *PTN*, *CNTNAP2*, *ELN*, *HSPB1*, *SEMA3E*, and *COL1A2*, were upregulated in the 11q-deleted group (n=8). In the same group of tumors, *CD44* was the top upregulated gene on 11p. Interestingly, on 11q, several tumor suppressor genes and genes encoding DNA-binding proteins involved in DNA repair and negative regulation of transcription were downregulated in the 11q-deleted tumors: *C11orf30*, *RSF1*, *CREBZF*, *FAT3*, *MRE11A*, *ATM*, *CADM1*, *MLL*, *H2AFX*, *TBRG1*, and *CHK1*.

## Discussion

In this report, we describe the DNA copy number profiles of a consecutive series of neuroblastomas that were selected on the basis of unfavorable characteristics. The findings revealed considerable genetic heterogeneity within this clinically troublesome group, which was particularly evident when comparing tumors with MNA to those with segmental 11q deletions. With few exceptions, MNA and segmental 11q loss were mutually exclusive and defined two genetic subgroups of equal size that comprised more than three-quarters of the total samples. Such genetic dichotomy of advanced neuroblastoma has been well described previously [2,7,8,11,15] and both MNA and segmental 11q loss are included in the current INRG algorithm for pretreatment stratification of risk [10]. Less predictably, we also observed a clear clinical difference between these two genetic subgroups in relation to age: MNA tumors affected the youngest children of the series. It is surprising that this age dependence with respect to the tumor genetics of neuroblastoma has not received much scientific attention previously, although mentioned in several previous studies [9,11,15,25]. In view of the relatively moderate size of the present tumor series, it was important that we were able to confirm a generally low age at diagnosis for children with MNA tumors using independent data from the Swedish Childhood Cancer Registry; these data argued clearly against a bias in the present material. We conclude from the present findings that unfavorable neuroblastomas are predominantly of the MNA type when diagnosed under the age of 2 years, whereas tumors with loss of 11q and other genetic variants predominate after 3.5 years of age.

As the Swedish Childhood Cancer Registry, due to lack of records, could not be used to verify the older age at diagnosis for children with 11q-deleted tumors we searched the literature for this information: Spitz et al. [9] reported on segmental 11q deletions from a cohort of 611 neuroblastomas, found in 159 tumors. The median age at diagnosis of these 11q-deleted tumors was 3–5 years, constituting 59 percent of the tumors of this age range. Michels et al. [11] reported 48 and 28 months as median ages at diagnosis for ten 11q-deleted and 22 MNA tumors, respectively. In a meta study by Vandesompele et al. [25] poor risk neuroblastomas were separated into two genetic “clusters”: The median age of 45 children belonging to the 11q deletion “cluster” was 41 months, although notably only 30 of the tumors were actually 11q deleted. The corresponding figures for 74 children belonging to the MNA “cluster” was 26.9 months median age with actual MNA seen in 51 tumors. Finally, Carén et al. [15] reported a median age of 42 months for 21 children with 11q-deleted tumors (four of which were common with our study) compared to 21 months for 37 children with MNA tumors (seven in common with our study). Together, these data consistently support an age difference between the two groups, although a certain bias towards older ages for 11q-deleted tumors in the present study is observed (58.5 months median age).

Another difference that was observed between MNA and 11q-deleted tumors was the higher number of SCAs in the later group of tumors and a dependence of their prevalence on age, both of which imply a chromosomal instability phenotype, as suggested previously by Carén et al. [15]. Future analyses using larger sample sizes, including multiple tumor specimens (synchronous and metachronous), might be helpful in confirming such chromosomal instability. Also, deep sequencing might shed additional light on differences in genomic integrity at the DNA level among subsets of neuroblastoma. A recent report [26], based on whole-genome sequencing of 87 neuroblastomas of all stages, showed that the frequency of somatic amino-acid-changing mutations strongly correlated to tumor stage, survival, and age at diagnosis. However, no difference in frequency of such mutations was detected when comparing MNA tumors to high-stage non-MNA tumors. Hence, this aformentioned study reported a general age dependence of amino-acid-changing mutations in neuroblastoma, albeit the specific issue of DNA instability in tumors with deleted 11q, as compared to MNA tumors, was not addressed.

On the basis of the profile subtypes that were observed in the presently relatively limited series of tumors, and in view of previous data by others, we speculate on the existence of four genetic routes for the genesis of aggressive neuroblastoma:

### The MNA route

MNA neuroblastomas seem to fit into a model of rapid tumor evolution that involves only a few genetic events, which transform the progenitor cells into highly proliferative and primary metastatic tumor cells in a straightforward fashion. The likelihood of such few events to take place would, logically, be more proportional to the pool of cells of origin than for tumors requiring a larger sequence of genetic hits (e.g. 11q deleted tumors), hence explaining the early in life appearance of MNA tumors.

### The 11q route

It appears that the pathogenesis of tumors with segmental 11q loss accords with the traditional genetic model for adult cancer, which predicts a micro-evolutionary process of cancer development, with successive genetic hits affecting key cellular functions [27]. Acquisition of chromosomal instability might be fundamental in this process. Our analysis of differential gene expression in 11q-deleted tumors *vs.* MNA neuroblastomas pinpointed some candidate tumor suppressor/DNA repair genes within the region of 11q that is deleted, such as *ATM*, *MLL*, *H2AFX*, and *CHK1*. Recently, the importance of aberrant gene expression within this region was underscored by the finding that decreased expression of genes within the region of interest at 11q was observed only in those 11q-deleted tumors with an aggressive clinical phenotype [28]. It is noteworthy, in this context, that a subgroup of 11q-deleted tumors with more favorable clinical characteristics has been described [28,29]. Apart from not showing a disproportionate downregulation of the expression of genes of the deleted region, such tumors were also reported to differ from unfavourable 11q-deleted tumors regarding microRNA expression profiles [29] and by having less SCAs [28]. It is therefore possible that the here suggested 11q route is not an important element in the pathogenesis of less aggressive tumors with 11q deletions. Evidently, our statistics on the present 11q-^notMNA^ tumors represents the unfavorable type of 11q-deleted neuroblastoma since there was only one survivor in this group. It is noticeable in this context that our inclusion criteria would discriminate against tumors of the more favorable subtype.

### The tumor progression route

A third possibility in the evolution of aggressive neuroblastoma is progression from lower-risk tumors [13]. Although progression from a low-risk lesion to an aggressive tumor is considered in general to be a rare event, we think that three of the nonMNA/non11q deleted tumors in the present study might have evolved in this way. Two tumors showed genetic similarity to low-risk tumors in that they showed whole chromosome aberrations for more than half of the chromosomes and only few segmental gains or losses. It appears particularly likely that a third tumor (ID 226) was derived from a lower-risk tumor: on diagnosis at 7.5 years of age, it was revealed that a chest X-ray taken 6 years earlier already then showed the primary tumor. Genetically, the tumor had much in common with 11q deleted tumors, but 11q was intact.

### Alternative amplicon driven routes

An unusual genetic variant of the present study was a tumor with multiple amplicons on 11q and 12q. There are other examples in the literature of neuroblastoma cell lines and tumors with very similar amplified regions at 11q13 [11,25] or 12q13–q15 [11,21,30], respectively, and in two tumors synchronous amplification on both chromosomes have been reported [11,31,32], as in the present case. *CCND1*, *IGHMBP2*, *ORAOV1*, *FGF4* on chromosome 11q13, and *CDK4* and *MDM2* on 12q13-15 are interesting oncogenes in this context. Generally, neuroblastomas with 11q13 amplicons were reported to be clinically aggressive and associated with an older age at diagnosis compared to children with MNA tumors of the present study. However, those 11q13 amplified neuroblastomas which also displayed MNA were diagnosed at earlier ages [11]. Clinical information on 12q amplified neuroblastoma is sparse in the literature but indicates advanced disease [30,33,34]. MNA may occur also in 12q amplified neuroblastoma [21,30,31]. Amplicons of other chromosomal regions are described in single cases of poor outcome neuroblastoma, involving 16q.21, 4q.33, 6p12-21 [25], 5q33.3 [11], and the *MYC* containing 8q24 region [26]. Hence, with the support of these previously published data, it seems that our case harboring chromosome 11 and 12 amplifications is not totally unique and implies that such and other non-*MYCN* amplifications may underlie a minor subset of aggressive neuroblastomas.

## Conclusions

**Figure 6:**
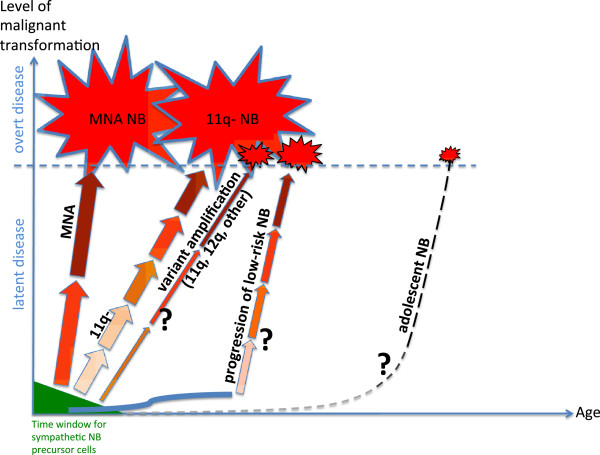
**A proposed model for the age dependence of unfavorable neuroblastoma. **The model builds on the assumption of an early common cellular origin of all neuroblastomas. Depending on genetic subtype the respective tumorigenic hits differ in type, number and degree of malignant effect - most evident when comparing MNA and 11q- tumors. As tumors with alternative amplifications, putative low-to-high-risk progression, and adolescent presentation are poorly represented in the present study and in the literature these routes for tumor development are largely speculative, indicated by question marks. *Abbreviation*: NB: neuroblastoma; *Symbols*: Arrows: tumorigenic hits; Darkness of arrow: degree of malignant transformation; Arrow width/size of symbol for clinical disease: relative frequency of tumor subtype; Solid curved blue line: low-risk neuroblastoma development.

On the basis of these categories, we suggest the following metaphor for the genesis of unfavorable neuroblastoma: MNA, segmental deletion of 11q, and low-risk precursor lesions provide an elevator, a staircase, and a first step of a staircase, respectively, towards unfavorable neuroblastoma. This metaphor builds on the notion that the first steps in tumor development take place during a common early developmental phase when the immature cells that are the source of neuroblastoma are still present in the sympathetic nervous system, whereas subsequent tumorigenic events differ in malignant effect between the genetic routes. Our speculations on main routes for the pathogenesis of unfavourable neuroblastoma are summarized in Figure 6, showing the four mentioned routes and adding also adolescent neuroblastoma as a separate, but still obscure, route. The present delineation of genetic subsets of unfavorable neuroblastoma might have therapeutic implications. It would seem that MNA tumors are more proliferative, which calls for a dose-intensive treatment, and the relatively “clean” genetics of MNA neuroblastomas would appear to make them suitable for tailored treatments that target the functions of MYCN, e.g. apoptosis [35]. Novel therapeutic strategies specifically for 11q-deleted neuroblastoma are urgently needed, and the identification of novel subsets of neuroblastomas with non-*MYCN* amplifications may also have therapeutic consequences, but these will have to await more conclusive information on the genetic and clinical homogeneity of such tumors. Finally, the possibility of a transition from low- to high-risk disease in some cases may have implications for the monitoring of low-risk rest tumors.

## Abbreviations

Adr: Adrenal; BAC: Bacterial artificial chromosome; CGH: Comparative genomic hybridization; DOD: Dead of disease; INRGSS: International Neuroblastoma Risk Group Staging System; MNA: *MYCN* amplification; NED: No evidence of disease; NB: Neuroblastoma; SCA: Segmental chromosomal aberration; SD: Stable disease; Th: Thoracic; WCA: Whole chromosomal aberration; 11q-: Segmental deletion on 11q.

## Competing interests

None of the authors have declared any financial or non-financial competing interests.

## Authors’ contributions

CC performed DNA preparations and BAC array analyses, compiled the data, and participated in drafting the manuscript. TM was responsible for Affymetrix SNP array verification analyses and contributed with critical revision of the manuscript. JS contributed with important methodological assistance with the BAC array analyses and critical reading of the manuscript. CT contributed with the data from the Swedish Childhood Cancer Registry. PK contributed with tumor materials and clinical data and conceptually in the drafting of the manuscript, JD had chief responsibility for the development and use of the BAC array platform and contributed with critical revision of the manuscript. TDS had main responsibility for the design and realization of the study, including drafting of the manuscript. FH conceived of the study, participated in its design, was responsible for collecting tumor samples and clinical data throughout the years, and had main responsibility for the drafting of the manuscript. All authors read and approved the final manuscript.

## Authors’ information

CC held a postdoc research position at the Department of Immunology, Genetics and Pathology, Uppsala University during the work. TM is a Professor of tumor genetics at the Department of Clinical Genetics, University of Gothenburg and a senior researcher on neuroblastoma. JS was a PhD student at the Department of Immunology, Genetics and Pathology, Uppsala University involved in the development of the BAC array platform and later holds a postdoc position at the Department of Oncology-Pathology, Karolinska Institutet. CT is an MD, PhD, specialist in pediatric oncology and a senior researcher on neuroblastoma at Karolinska Institutet. PK is an MD, PhD, and specialist in pediatric oncology with national responsibilities for neuroblastoma treatment, and senior researcher on neuroblastoma at Karolinska Institutet. JD is a professor of experimental pathology at the Department of Immunology, Genetics and Pathology, Uppsala University. TDS is an associate Professor at the Department of Oncology-Pathology, Karolinska Institutet and formerly at the Department of Immunology, Genetics and Pathology, Uppsala University. FH is an MD, PhD, and specialist in pediatric oncology and senior researcher on neuroblastoma at the Department of Immunology, Genetics and Pathology and Women’s and Children’s Health, Uppsala University.

## Pre-publication history

The pre-publication history for this paper can be accessed here:

http://www.biomedcentral.com/1471-2407/13/231/prepub

## Supplementary Material

Additional file 1: Figure S1 Schematic representation of genetic profiles for neuroblastoma samples included in the study (n=34). Deleted and gained regions are represented by red and green bars, respectively. Cases are arranged according to genetic subgroup. Click here for file

Additional file 2: Figure S2 Novel amplicons in association with MNA on 2p: 32K whole-genome array data are shown from: (A) the primary tumor of case ID55; (B) cell line U2715, which was established from this tumor. Click here for file

## References

[B1] Brodeur GM Neuroblastoma: biological insights into a clinical enigma Nat Rev Cancer 2003 3 3 203 216 10.1038/nrc1014 12612655

[B2] Maris JM Recent advances in neuroblastoma N Engl J Med 2010 362 23 2202 2211 10.1056/NEJMra0804577 20558371 PMC3306838

[B3] Park JR Eggert A Caron H Neuroblastoma: biology, prognosis, and treatment Hematol Oncol Clin North Am 2010 24 1 65 86 10.1016/j.hoc.2009.11.011 20113896

[B4] Franks LM Bollen A Seeger RC Stram DO Matthay KK Neuroblastoma in adults and adolescents: an indolent course with poor survival Cancer 1997 79 10 2028 2035 10.1002/(SICI)1097-0142(19970515)79:10<2028::AID-CNCR26>3.0.CO;2-V 9149032

[B5] Polishchuk AL Dubois SG Haas-Kogan D Hawkins R Matthay KK Response, survival, and toxicity after iodine-131-metaiodobenzylguanidine therapy for neuroblastoma in preadolescents, adolescents, and adults Cancer 2011 117 18 4286 4293 10.1002/cncr.25987 21387264 PMC3125487

[B6] Brodeur GM Seeger RC Schwab M Varmus HE Bishop JM Amplification of N-myc in untreated human neuroblastomas correlates with advanced disease stage Science 1984 224 4653 1121 1124 10.1126/science.6719137 6719137

[B7] Janoueix-Lerosey I Schleiermacher G Michels E Mosseri V Ribeiro A Lequin D Vermeulen J Couturier J Peuchmaur M Valent A Overall genomic pattern is a predictor of outcome in neuroblastoma J Clin Oncol 2009 27 7 1026 1033 10.1200/JCO.2008.16.0630 19171713

[B8] Attiyeh EF London WB Mosse YP Wang Q Winter C Khazi D McGrady PW Seeger RC Look AT Shimada H Chromosome 1p and 11q deletions and outcome in neuroblastoma N Engl J Med 2005 353 21 2243 2253 10.1056/NEJMoa052399 16306521

[B9] Spitz R Hero B Simon T Berthold F Loss in chromosome 11q identifies tumors with increased risk for metastatic relapses in localized and 4S neuroblastoma Clinical cancer research : an official journal of the American Association for Cancer Research 2006 12 11 Pt 1 3368 3373 16740759 10.1158/1078-0432.CCR-05-2495

[B10] Cohn SL Pearson AD London WB Monclair T Ambros PF Brodeur GM Faldum A Hero B Iehara T Machin D The international neuroblastoma risk group (INRG) classification system: an INRG task force report J Clin Oncol 2009 27 2 289 297 10.1200/JCO.2008.16.6785 19047291 PMC2650388

[B11] Michels E Vandesompele J De Preter K Hoebeeck J Vermeulen J Schramm A Molenaar JJ Menten B Marques B Stallings RL ArrayCGH-based classification of neuroblastoma into genomic subgroups Genes Chromosomes Cancer 2007 46 12 1098 1108 10.1002/gcc.20496 17823929

[B12] Mosse YP Diskin SJ Wasserman N Rinaldi K Attiyeh EF Cole K Jagannathan J Bhambhani K Winter C Maris JM Neuroblastomas have distinct genomic DNA profiles that predict clinical phenotype and regional gene expression Genes Chromosomes Cancer 2007 46 10 936 949 10.1002/gcc.20477 17647283

[B13] Schleiermacher G Janoueix-Lerosey I Ribeiro A Klijanienko J Couturier J Pierron G Mosseri V Valent A Auger N Plantaz D Accumulation of segmental alterations determines progression in neuroblastoma J Clin Oncol 2010 28 19 3122 3130 10.1200/JCO.2009.26.7955 20516441

[B14] Hedborg F Lindgren PG Johansson I Kogner P Samuelsson BO Bekassy AN Olsen L Kreuger A Pahlman S N-myc gene amplification in neuroblastoma: a clinical approach using ultrasound guided cutting needle biopsies collected at diagnosis Med Pediatr Oncol 1992 20 4 292 300 10.1002/mpo.2950200405 1608350

[B15] Caren H Kryh H Nethander M Sjoberg RM Trager C Nilsson S Abrahamsson J Kogner P Martinsson T High-risk neuroblastoma tumors with 11q-deletion display a poor prognostic, chromosome instability phenotype with later onset Proc Natl Acad Sci U S A 2010 107 9 4323 4328 10.1073/pnas.0910684107 20145112 PMC2840092

[B16] Diaz De Stahl T Sandgren J Piotrowski A Nord H Andersson R Menzel U Bogdan A Thuresson AC Poplawski A Von Tell D Profiling of copy number variations (CNVs) in healthy individuals from three ethnic groups using a human genome 32 K BAC-clone-based array Hum Mutat 2008 29 3 398 408 10.1002/humu.20659 18058796

[B17] Sambrook JFE Maniatis T Molecular Cloning; a Laboratory Manual 1989 Cold Spring Harbor, NY: Cold Spring Harbor Laboratory Press

[B18] Ameur A Yankovski V Enroth S Spjuth O Komorowski J The LCB data warehouse Bioinformatics 2006 22 8 1024 1026 10.1093/bioinformatics/btl036 16455749

[B19] Andersson R Bruder CE Piotrowski A Menzel U Nord H Sandgren J Hvidsten TR Diaz de Stahl T Dumanski JP Komorowski J A segmental maximum a posteriori approach to genome-wide copy number profiling Bioinformatics 2008 24 6 751 758 10.1093/bioinformatics/btn003 18204059

[B20] Yang YH Dudoit S Luu P Lin DM Peng V Ngai J Speed TP Normalization for cDNA microarray data: a robust composite method addressing single and multiple slide systematic variation Nucleic Acids Res 2002 30 4 e15 10.1093/nar/30.4.e15 11842121 PMC100354

[B21] Caren H Erichsen J Olsson L Enerback C Sjoberg RM Abrahamsson J Kogner P Martinsson T High-resolution array copy number analyses for detection of deletion, gain, amplification and copy-neutral LOH in primary neuroblastoma tumors: four cases of homozygous deletions of the CDKN2A gene BMC Genomics 2008 9 353 10.1186/1471-2164-9-353 18664255 PMC2527340

[B22] Lastowska M Viprey V Santibanez-Koref M Wappler I Peters H Cullinane C Roberts P Hall AG Tweddle DA Pearson AD Identification of candidate genes involved in neuroblastoma progression by combining genomic and expression microarrays with survival data Oncogene 2007 26 53 7432 7444 10.1038/sj.onc.1210552 17533364

[B23] Smyth GK Linear models and empirical bayes methods for assessing differential expression in microarray experiments Stat Appl Genet Mol Biol 2004 3 Article3 16646809 10.2202/1544-6115.1027

[B24] Benjamini YHY Controlling the false discovery rate: a practical and powerful approach to multiple testing J R Stat Soc 1995 Ser B 57 289 300

[B25] Vandesompele J Baudis M De Preter K Van Roy N Ambros P Bown N Brinkschmidt C Christiansen H Combaret V Lastowska M Unequivocal delineation of clinicogenetic subgroups and development of a new model for improved outcome prediction in neuroblastoma Journal of clinical oncology : official journal of the American Society of Clinical Oncology 2005 23 10 2280 2299 10.1200/JCO.2005.06.104 15800319

[B26] Molenaar JJ Koster J Zwijnenburg DA van Sluis P Valentijn LJ van der Ploeg I Hamdi M van Nes J Westerman BA van Arkel J Sequencing of neuroblastoma identifies chromothripsis and defects in neuritogenesis genes Nature 2012 483 7391 589 593 10.1038/nature10910 22367537

[B27] Hanahan D Weinberg RA Hallmarks of cancer: the next generation Cell 2011 144 5 646 674 10.1016/j.cell.2011.02.013 21376230

[B28] Fischer M Bauer T Oberthur A Hero B Theissen J Ehrich M Spitz R Eils R Westermann F Brors B Integrated genomic profiling identifies two distinct molecular subtypes with divergent outcome in neuroblastoma with loss of chromosome 11q Oncogene 2010 29 6 865 875 10.1038/onc.2009.390 19901960

[B29] Buckley PG Alcock L Bryan K Bray I Schulte JH Schramm A Eggert A Mestdagh P De Preter K Vandesompele J Chromosomal and microRNA expression patterns reveal biologically distinct subgroups of 11q- neuroblastoma Clin Cancer Res 2010 16 11 2971 2978 10.1158/1078-0432.CCR-09-3215 20406844 PMC2880207

[B30] Chen QR Bilke S Wei JS Whiteford CC Cenacchi N Krasnoselsky AL Greer BT Son CG Westermann F Berthold F cDNA array-CGH profiling identifies genomic alterations specific to stage and MYCN-amplification in neuroblastoma BMC Genomics 2004 5 70 10.1186/1471-2164-5-70 15380028 PMC520814

[B31] Carr J Bown NP Case MC Hall AG Lunec J Tweddle DA High-resolution analysis of allelic imbalance in neuroblastoma cell lines by single nucleotide polymorphism arrays Cancer Genet Cytogenet 2007 172 2 127 138 10.1016/j.cancergencyto.2006.08.012 17213021

[B32] Corvi R Savelyeva L Amler L Handgretinger R Schwab M Cytogenetic evolution of MYCN and MDM2 amplification in the neuroblastoma LS tumour and its cell line Eur J Cancer 1995 31A 4 520 523 7576957 10.1016/0959-8049(95)00031-d

[B33] Rudolph G Schilbach-Stuckle K Handgretinger R Kaiser P Hameister H Cytogenetic and molecular characterization of a newly established neuroblastoma cell line LS Hum Genet 1991 86 6 562 566 2026421 10.1007/BF00201542

[B34] Su WT Alaminos M Mora J Cheung NK La Quaglia MP Gerald WL Positional gene expression analysis identifies 12q overexpression and amplification in a subset of neuroblastomas Cancer Genet Cytogenet 2004 154 2 131 137 10.1016/j.cancergencyto.2004.02.009 15474148

[B35] Amati B Littlewood TD Evan GI Land H The c-Myc protein induces cell cycle progression and apoptosis through dimerization with Max EMBO J 1993 12 13 5083 5087 8262051 10.1002/j.1460-2075.1993.tb06202.x PMC413769

