# Supplementary figures and images for: Age dependence of tumor genetics in unfavorable neuroblastoma: arrayCGH profiles of 34 consecutive cases, using a Swedish 25-year neuroblastoma cohort for validation
Source: BMC Cancer. 2013 May 9;13:231. doi: 10.1186/1471-2407-13-231 (PMC3664071; doi:10.1186/1471-2407-13-231)

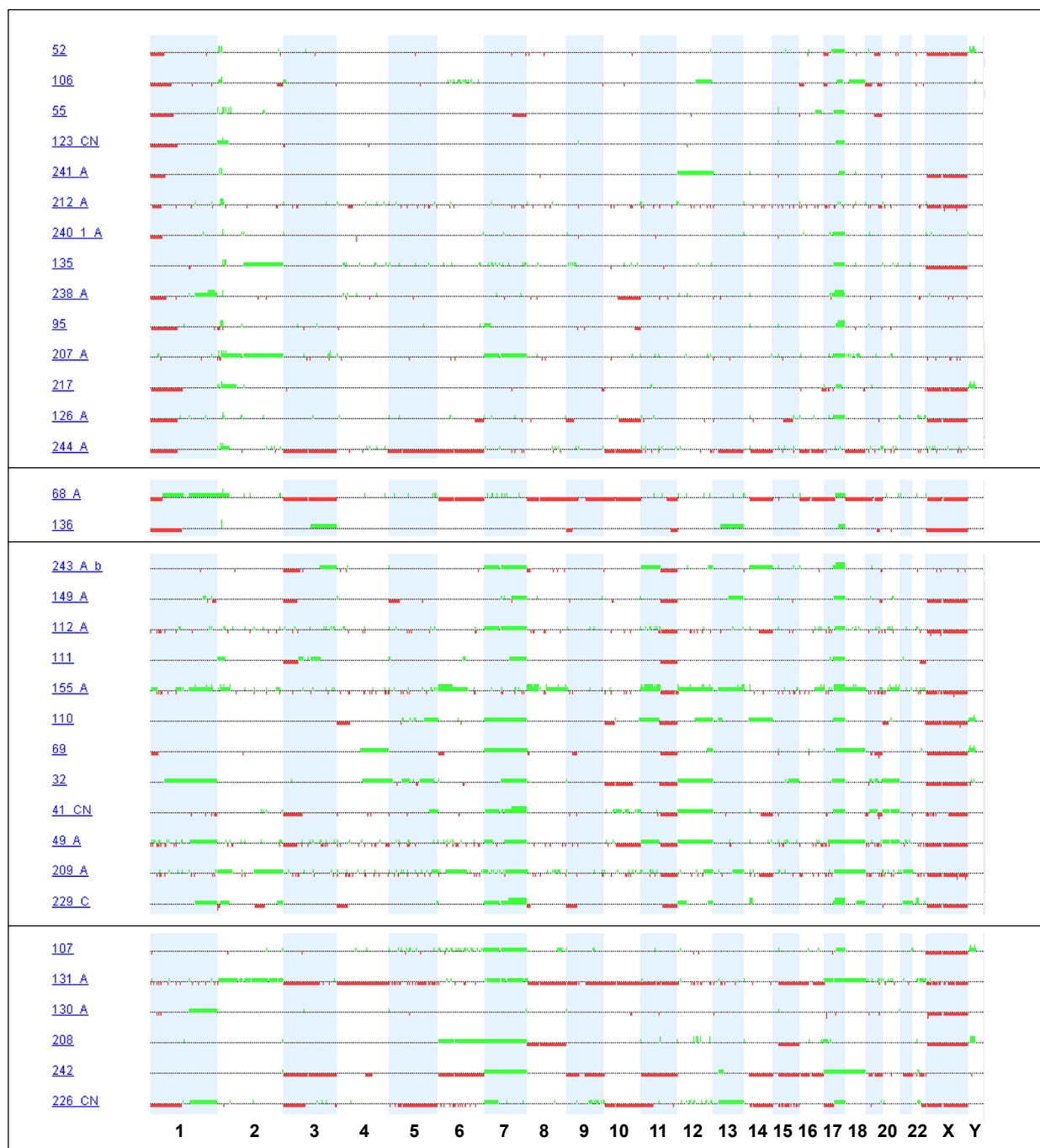

Supplement: Additional file 1: Figure S1 — Schematic representation of genetic profiles for neuroblastoma samples included in the study (n=34). Deleted and gained regions are represented by red and green bars, respectively. Cases are arranged according to genetic subgroup. [file 1471-2407-13-231-S1.pdf]

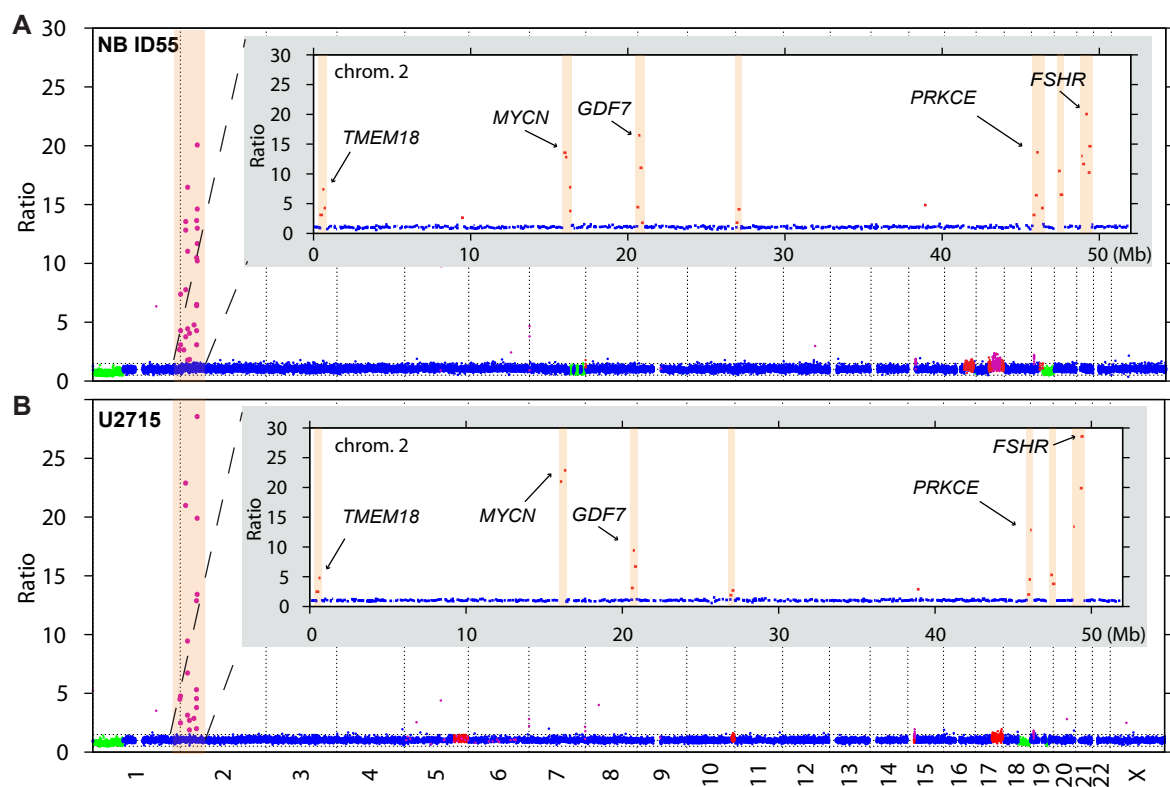

Supplement: Additional file 2: Figure S2 — Novel amplicons in association with MNA on 2p: 32K whole-genome array data are shown from: (A) the primary tumor of case ID55; (B) cell line U2715, which was established from this tumor. [file 1471-2407-13-231-S2.pdf]
